# Analysis of Low Cancer Mortality Rates in the Wine Regions of Tokaj and Balaton in Hungary

**DOI:** 10.3390/ijerph17186759

**Published:** 2020-09-16

**Authors:** Sándor Sipka, János Nagy, Péter Sipka, Judit Kocsis, Judit Tóth, Péter Árkosy, Zsolt Horváth

**Affiliations:** 1 Department of Nutrition, Faculty of Medicine, University of Debrecen, 4032 Debrecen, Hungary; 2 Department of Oncology, Faculty of Medicine, University of Debrecen, 4032 Debrecen, Hungary; nagyjanos@unideb.hu (J.N.); judit.toth@med.unideb.hu (J.T.); arkosy.peter@med.unideb.hu (P.Á.); 3 Department of Labor Law, Faculty of Law, University of Debrecen, 4028 Debrecen, Hungary; iroda@drsipka.hu; 4 Bács-Kiskun County Hospital, Centre of Oncoradiology, Kecskemét, 6000 Kecskemét, Hungary; kocsisjucidr@gmail.com (J.K.); zsodicsom@gmail.com (Z.H.)

**Keywords:** age-adjusted death rate, cancer, wines of Balaton, wines of Tokaj

## Abstract

The age-adjusted death rates (AADRs) due to cancers were investigated in two historical regions of white wines (Tokaj and Balaton) and in Hódmezővásárhely (HMV) as a control territory in Hungary between 2000 and 2010 evaluating 111,910 persons. The results of AADRs due to the eight most frequent types/gastrointestinal cancers were as follows: Tokaj 2120/664, Balaton: 2417/824, HMV: 2770/821, nationwide: 2773/887. The values found in Tokaj and Balaton regions were significantly less than those of HMV and nationwide. However, the least values were found in Tokaj. This Tokaj-related strong difference was not found among the regions in the case of young populations with hematological diseases but only in the older people who have been consuming their wines for decades. Supposedly, this wine-specific anti-cancer phenomenon could be related to the chemical differences existing in the two types of white wines, namely, to the pro-oxidant molecules of Tokaj wines derived from *Botrytis cinerea*. The roles of red meat consumption, hardness of drinking water, mineral content of soil, and socioeconomic status were negligible. It should be stressed that these data are valid only for these populations, for this period. Noteworthily, the different types of wines may have different effects on mortality rates during long-lasting consumptions.

## 1. Introduction

In the present work, we compared the age-adjusted mortality rates (AADRs) due to cancers in two traditional white wine regions of Hungary and in a control, non-wine region in a large population who died between 2000 and 2011. We chose this form of study for two reasons: (a) While a very great number of observations were already published on the effects of red wine consumption on cancers mostly due to Mediterranean diets (including vegetables, fruits, and red wines) [1,2], surprisingly and inversely, no remarkable articles could be found on the topic of “white wines and cancers”, especially derived from a large population [1]; (b) The obvious chemical differences between the more than 20,000 molecules of red and white wines [3] hinder the comparability and correct evaluations of their eventually different biological effects out of the ethyl-alcohol contents. 

Thus, for the sake of better comparability, we aimed to study now the cancer mortality rates: (a) on a large population and (b) in the two greatest historical white wine territories of Hungary, in the regions of Tokaj and Balaton. The control field was Hódmezővásárhely (HMV), which is not a wine region. The values of age-adjusted death rates (AADRs) due to the most frequent eight types of cancers were compared between 2000 and 2010 using the data of 111,910 persons. As the increased consumption of red and processed meats might have a positive relation with colorectal carcinogenesis [4], we collected data on this nutrition habit of inhabitants also (meat kg/person/year). For social comparability among the regions, the indices of socioeconomic deprivation (ID) were determined [5]. As these data were derived from the results of the national census carried out in 2011, any significant changes in lifestyle between 2000 and 2010 could be investigated by statistical analysis, proving the comparability of regions with regard to socioeconomic aspects. Since, the hardness of drinking waters also might have some effects on gastric cancer mortality [6], the values of total hardness of drinking waters (CaO mg/L) were compared. In addition, the 14 mineral elements of soils were investigated, too. As the studies on Mediterranean diet suggested some beneficial effects of vegetables and fruits on cancer prevention [1,2], we collected data characterizing the populations from this nutritional point of view also.

## 2. Methods

### 2.1. The Types of Wines and the Settlements of Territories 

The subjects of this study belonged to three regions. The first two regions were wine producing and the third one was control: A. Tokaj: with special, mostly *Botrytis cinerea-*related local types of “white” wines, named as “Tokaj wines”; B: Balaton: with mostly commercial “white” wines; C: Hódmezővásárhely (HMV) was not a wine region. The data were derived from 11 urban settlements of Tokaj and Balaton regions and from the control city, HMV.

### 2.2. Age-Adjusted Death Rates

The data of persons who died in Hungary during the studied 11 years long period of 2000–2010 were provided by the Central Bureau of Public and Electronic Administration and the Hungarian Central Statistical Office (HCSO). The data were grouped according to settlements, sex, and diagnosis. The cancer diagnoses were classified according to The International Statistical Classification of Diseases and Related Health Problems (ICD). We calculated the age-adjusted death rates (AADR values) for 100,000 subjects in every region for the whole period. In this form of calculation, “age” as a risk factor for cancers was excluded from the study. 

“AADR value” = total number of dead persons due to cancer/100,000 inhabitants/11 years.

### 2.3. The Measurements of the Indicators of Hardness of Drinking Waters and the Mineral Element Contents of Soils 

We used only the summarized data of the total hardness (CaO mg/L) of drinking waters for each region collected from the National Institutes of Health (Budapest). The data on the mineral element contents of soils were derived from National Institute of Food Safety (Budapest).

### 2.4. Calculation of the Index of Socioeconomic Deprivation (ID) 

The comparison of socioeconomic status occurred by the calculation of an index of deprivation (ID) [5] using the data from HCSO gained by the latest national census in 2011. Deprivation means retardation or underdevelopment. A negative sign indicates a better social state. It was important that we tested only the comparable urban populations representing living standards higher than the average of Hungary (ID = 0.00). They all showed negative ID values with small numeric differences. The values of ID were calculated from data of HCSO using seven indicators. The levels of health care service, medical background, consumption of medicines, and the basic conditions for diagnosis and treatment of cancers were similar in the three regions. 

### 2.5. The Red Meat Consumption

The data of red meat consumption (kg/person/year) were derived from the National Board for Flesh of Beef and Pork collected first and last in Hungary in 2007, just in the period of our study. These results did not contain the data of smoke-cured meats (sausage, salami, ham, etc.) and the flesh from poultry.

### 2.6. The Consumption of Vegetables and Fruits

Concerning the consumptions of vegetables and fruits (without potato) in the various regions of Hungary, we used the data of HCSO derived from 2009 showing the averages of expenses/person expressed in the percent of nationwide values. 

### 2.7. Statistical Analysis

The AADR values calculated annually during the 11 years were summarized and then adjusted to the population sizes of the three different regions [7] and to the nationwide values. The significant differences between the various cancer mortality values were calculated by chi-square test. The IBM SPSS ver. 24 program (IBM Corp, Armonk, NY, USA) was used for the calculations.

## 3. Results

### 3.1. The Number of Population, Index of Deprivation, Consumption of Red Meat as Well as Vegetable/Fruit and Total Hardness of Drinking Waters in the Three Regions between 2000 and 2010

The numbers of populations were larger than 30,000 in each region as follows: Tokaj: 33,917; Balaton: 30,833; HMV: 47,160. In the 11 urban settlements, the socioeconomic status was better than the average national living standard (0.00) demonstrated by the “negative signs” of indices of ID: Tokaj: −0.36; Balaton: −1.22; HMV: −0.43. Thus, despite of small differences, the three populations were comparable from a socioeconomic aspect. The amounts of red meat consumption showed the following results: Tokaj: 16.15; Balaton 18.65; HMV: 24.35 (kg/person/year). The averages of vegetable/fruit consumptions expressed as percent of nationwide values did not show remarkable differences as follows: Tokaj: 85%; Balaton: 98%; HMV: 105%. The results on the hardness of drinking waters and cancer mortality values did not correlate to each other as could be seen from the following results: in Tokaj and HMV the drinking waters were of similar “soft character” (138.60 and 81.90 CaO mg/L), but the AADR values for gastrointestinal cancers were significantly different (664 versus 821, *p* = 0.009). In addition, the Balaton region with drinking waters of “hard character” (249.20 CaO mg/L) showed almost the same AADR value as HMV (824 and 821). The contents of mineral elements in soils are not presented in detail because they also do not contain any relevant data on cancers. The main parameters are presented in Table 1.

### 3.2. The AADR Values of Various Types of Cancers/Malignancies in the Three Regions and Nationwide between 2000 and 2010

In Table 2 are grouped the AADR values due to the most frequent eight types of malignancies signed as “all cancers” in the three territories and compared to the nationwide values. Regarding “all cancers” the AADR values of Tokaj (2120, *p* < 0.0005) and Balaton (2425, *p* = 0.003) were significantly lower than that of HMV (2771) and nationwide (2773). Furthermore, the AADR value of Tokaj was still significantly less than that of Balaton (2120 versus 2425, *p* = 0.013). The AADR values of various types of cancers/malignancies reflected three mostly significant tendencies: (a) the lowest values for 6/8 of cancers were found in Tokaj as follows: gastrointestinal (664 versus 824, 821, 887; *p* = 0.018, *p* = 0.009, *p* = 0.0005), female breast (Tokaj: 128 versus Balaton: 140, *p* = 0.013), female genital organs (133), male genital organs (71), urinary tract (72). However, concerning the malignancies of lymphoid and hematological organs that appeared in childhood, two comparisons took place. Firstly, we tested the total mortality rates, which showed the significantly least value in Tokaj compared to that nationwide (102 versus 155, *p* = 0.015), Balaton (148), and HMV (183). Then, among the persons who died under the age of 25 years, no significant differences were found as follows: Tokaj: 6.0, Balaton: 7.9, nationwide: 6.1. Regarding these facts, the potential anti-cancer effects of Tokaj wines seemed to appear only in the later phase of life when the wine consumption could already have become a habit of an older person. (b) The value of lip, mouth, and pharyngeal cancers in Tokaj (167) was higher than that of Balaton (146), HMV (105), and the nationwide (155). (c) In HMV, the AADR value was extremely high concerning respiratory cancers (831 versus 488 and 557, *p* < 0.0005). It was of note that the AADR values of female genital cancers did not reflect any significant differences in the regions: Tokaj: 133; Balaton: 180; HMV: 139; nationwide: 141. On the other hand, the AADR values of female breast cancer showed a significant difference, as follows: 128 (Tokaj) versus 185 (nationwide) (*p* = 0.013).

## 4. Discussion

In the wine regions of Tokaj and Balaton, the AADR values due to “all cancers” were significantly lower than those in HMV and nationwide. However, the AADR value was still significantly less in Tokaj than that in the region of Balaton, which was also dominantly a “white wine” territory. This striking difference suggests that considerable chemical differences can exist between the white wines of Tokaj and Balaton. The AADR values of the most common eight types of cancers form three groups: (a) six of them showed the lowest AADR values in Tokaj; (b) concerning the cancers of lip, oral cavity, and pharynx, Tokaj presented the highest AADR value; (c) HMV showed the highest mortality rate of respiratory cancers. It is of note however, that comparing the AADR values of malignant lymphoid and hematological diseases already existing in childhood, there were differences in the whole population and in the persons who died under the age of 25 years. The significantly low rate of these malignancies in the elder population of Tokaj suggests the first indirect proof for the wine- and region-specific anti-cancer effects of these wines. As alcohol consumption may only reduce the risk of non-Hodgkin lymphoma and not that of others [8], the finding concerning the possible specific protective effects of Tokaj wines against most malignant diseases can be valid and considerable, but characteristic and indirectly proven only for these populations and for this period of time.

Since the values of red meat consumption show a tendency similar to that of the results of “all cancers” mortality rates: Tokaj < Balaton < HMV, two important remarks are required. First, the positive role of increased red meat consumption in colorectal cancers was observed in populations of such countries as USA and Hong Kong [4,9] consuming much more red meat/year than our people. In 2007, their values were more than 70 kg/person/year [10] in contrast to those of inhabitants in the three regions of our study: 16.15, 18.65, 24.35 kg/person/year, eating mostly poultry flesh. Second, just in the case of gastrointestinal cancers we can see that the AADR values of Balaton (824) and HMV (821) are almost the same, while the difference in the amounts of red meat consumption is relatively great: 18.65 versus 24.35 kg/person/year. They do not reflect the tendency of AADR at all. These results suggest that the differences in the chemical characters of the two white wines may have a greater impact on the cancer mortality rates than the values of red meat consumption (eating habit) in the various regions. Furthermore, the relatively small differences in the consumption of vegetables and fruits in the three populations (Tokaj: 85%, Balaton: 98%; HMV: 105%) did not reflect any connection to the values of AADR.

The data on indices of socioeconomic deprivation did not show any correlation with the mortality rates. In addition, the correlation between the total hardness of drinking waters and AADR was also not significant in the 11 urban settlements of the three territories despite the remarkable differences in the hardness of drinking waters (CaO mg/L): Tokaj: 138.60; Balaton: 249.20; and HMV: 81.90. The differences in the mineral content of soils were also negligible. However, it should be stressed very much that these findings were valid only for this period of time and these populations when these people were still drinking their own traditional wines and not beer or various imported mineral waters and soft drinks as they do in the later years. 

The comparability of wine and alcohol consumptions in the various regions was a critical point in the whole evaluation. As we worked with retrospective data (mortality rates) it was impossible to determine the individual wine, beer, and “pálinka” (national beverage) consumption as a total “alcoholic burden”. Therefore, we had to use an indirect marker to answer this crucial question. By great luck, we found data from 1994 on the occurrence of alcoholic liver cirrhosis in the various counties of Hungary reflecting the “pure ethyl-alcohol” consumption of inhabitants [11]. The cirrhosis values were almost similar in the Tokaj and Balaton regions, while it was less in HMV. The data were as follows: Tokaj: 400; Balaton: 506; HMV: 246; nationwide: 411. The difference between Tokaj and Balaton regions was statistically not significant: *p* = 0.053, but the differences concerning the AADR values e.g., for gastrointestinal cancers here showed a high level of significance: *p* = 0.018. Thus, as a matter of fact, the AADR values for the eight types of cancers were comparable from the aspect of “pure ethyl-alcohol consumption” (derived from wines, palinka, and beer) in the two wine regions, but the mortality rates were better in Tokaj. Furthermore, the AADR values for gastrointestinal cancers were almost the same in the regions of Balaton (824) and HMV (821), whereas the AADR for liver diseases was about twofold less in HMV (506 versus 246) suggesting that the ranges of “pure” alcohol consumption could not be a causative factor in the generation of these differences in cancer mortality. We were unable to collect exact data on the smoking habits. Although, the significantly high AADR value for respiratory cancers (831) in HMV suggested they could be the highest among the three regions. At the same time, the slightly higher value of respiratory cancer mortality in Tokaj (577) than in Balaton (488) clearly showed that the smoking habits probably could not play any role in causing the lowest value of AADR for all cancer observed in Tokaj.

However, it is necessary to comment on the scientific consensus declaring that alcohol drinking can cause several types of cancer [12]. Our data comparing the appearance of alcoholic liver cirrhosis as an indirect disease marker of “pure ethyl-alcohol consumption” also showed some similar tendency as in the two wine regions the values were close to each other (400,506), but in HMV it was less (246). However, it should be stressed that our results reflect mortality and not morbidity. It is one of the most important messages of this study that the special chemical differences of various wines should be better taken into consideration when their long lasting and complex biological effects are analyzed. Since, beside ethyl-alcohol, thousands of their other known or still undefined molecules can differently modify the survival rates of cancer patients. This opinion can be true for the wines of Tokaj and Balaton, as well. Last but not least, it is an important observation that “Beer and liquor had significant positive association with gastric cancer risk, while wine drinking would not increase gastric cancer risk” [13].

Therefore, the differences in the AADR values due to cancers could really derive from the chemical differences of the white wines dominating these two regions. We suppose that three chemical factors of Tokaj wines could certainly be involved in the significantly lower mortality rates of cancers in this territory than those in the regions of Balaton, HMV, and the values nationwide. They were as follows: (a) The group of mainly oxidative molecules (oxidases, H_2_O_2_, etc.) derived from *Botrytis cinerea* [14,15]. Although the *Botrytis* effects were dominant in the “Aszú” types of wines, more or less all sorts of grapes in this large area used to be infected by “noble yeast” at a certain extent because “Aszú” and “Szamorodni” production started here centuries before. The highly elevated pro-oxidant capability of *Botrytis* wines was firstly observed and described by Sipka and coworkers [16]; in addition, the anti-oxidant capacity of Aszú wines was found to be much less than that of red wines (not published observation, Sipka, 2019). (b) The remarkably higher amounts of iron (Fe^2+^ 6–18 mg/L) occurring in all these wines compared to those in red wines (2.0–3.0 mg/L) [3] could react with H_2_O_2_ (e.g., in the Fenton reaction) and could induce an increased production of reactive oxygen species (ROS) leading to apoptosis in colon cancer cells [17]. Additionally, H_2_O_2_ could stimulate the natural killer cells attacking the tumor cells [18]. (c) The wines affected by *Botrytis cinerea* might contain rather high amounts of spermidine (3–5 mg/L) and the anti-cancer effects of this molecule also could contribute to the decreased mortality of consumers [19,20].

Of course, a series of other molecules or chemical agents may still be discovered in the future explaining the special biological effects of these wines. However, it should be stressed that the surprising significant decreases in the mortality rates for the most frequent eight, and especially for the gastrointestinal cancers, could be observed only in Tokaj compared to the data of Balaton, HMV, and nationwide, which showed significantly higher values. Although, the results of Balaton were better (less) than those of HMV and nationwide, the results of Tokaj were even better from this aspect. These results drew the attention to the specific chemical components of wines concerning cancer mortality, while in the case of cardiovascular mortality, the hardness of drinking water and the socioeconomic deprivation had greater impacts than the types of dominant wines of a certain wine region [21].

## 5. Conclusions

This study presents significantly lower mortality rates due to cancers in the white wine region of Tokaj than that of Balaton, in addition to the values of nationwide and the non-wine region Hódmezővásárhely observed on a population larger 100,000 persons between 2000 and 2010. In this “Tokaj phenomenon”, supposedly, *Botrytis cinerea* could play a crucial role by the production of special and characteristic oxidative molecules. These observations are the first indirect proofs suggesting that the traditional wines of Tokaj might have some wine-type- and region-specific anti-cancer effects in this specific period of time, on a population still living their traditional way of life. Since then, a lot of changes have taken place not only in the lifestyle of people here but also in the wine producing technologies. However, these observations should be kept in mind suggesting that once (now) it was pointed out by scientific methods and on a large population that the real, traditional wines of Tokaj, in consequence of their exclusive *Botrytis* effects, potentially might have anti-cancer effects in the Tokaj region before 2010. 

At the same time, these results of Tokaj did not destroy the outstanding image of Balaton wines, as the cancer mortality rates of this region were also lower than that of the control non-wine region and nationwide. These results only mean that the anti-cancer potential of these wines might be less, but from other aspects they can be superior and more popular than the wines of Tokaj. Finally, these results may encourage wine-type-specific nutrition research, a trend of “typifying wine research”. 

## Figures and Tables

**Table 1:** Population, index of deprivation, consumption of red meat as well as vegetables/fruits and total hardness of drinking waters in the three regions between 2000 and 2010.

Parameters	Tokaj	Balaton	Hódmezővásárhely
Population (2010)	33,917	30,833	47,160
Index of Deprivation (2011)	−0.36	−1.22	−0.43
Red meat consumption (2007) (kg/person/year)	16.15	18.65	24.35
Expenses of vegetable/fruit consumption (2009, percent of nationwide average)	85	98	105
Total permanent hardness of drinking water (CaO mg/L)	138.60	249.20	81.90

**Table 2:** The age-adjusted death rate (AADR) values of various types of cancers/malignancies in the three regions and nationwide between 2000 and 2010.

Cancers/Malignancies	Tokaj	Balaton	HMV	Nationwide	Significance
Gastrointestinal (C15–C26)	1./664	3./824	2./821	887	1/2(0.009); 1/3(0.018)
Respiratory (C30–C39)	2./577	1./488	3./831	772	1/3(<0.0005); 2/3(<0.0005)
Lip, oral, pharyngeal (C00–C14)	3./167	2./146	1./105	155	1/nationwide (0.005)
Female genital (C51–C58)	1./133	180	2./139	141	-
Female breast (C50)	1./128	2./140	3./178	185	1/nationwide (0.013)
Lymphoid and hematological (C81–C96) (total)	1./102	2./148	3/183.	155	1/nationwide (0.015)
Lymphoid and hematological <25 years	6.0	7.9	18.5	6.1	-
Urinary (C64–C68)	1./72	2./106	3./145	123	-
Male genital (C60–C63)	1./71	3./99	2./95	91	-
All cancers	1./2120	2./2417	3./2771	2773	1/2(0.013); 1/3(<0.0005); 2/3(0.003);
